# Remission induction by Raising the dose of Remicade in RA (RRRR) study: Rationale and study protocol for a randomized controlled trial comparing for sustained clinical remission after discontinuation of infliximab in patients with rheumatoid arthritis

**DOI:** 10.1016/j.conctc.2017.08.007

**Published:** 2017-08-17

**Authors:** Koji Oba, Nao Horie, Norihiro Sato, Kazuyoshi Saito, Tsutomu Takeuchi, Tsuneyo Mimori, Nobuyuki Miyasaka, Takao Koike, Yoshiya Tanaka

**Affiliations:** a Interfaculty Initiative in Information Studies, Graduate School of Interdisciplinary Information Studies, The University of Tokyo, 7-3-1 Hongo, Bunkyo-ku, Tokyo, 113-0033 Japan; b Clinical Research and Medical Innovation Center, Hokkaido University Hospital, Kita 14, Nishi 5, Kita-ku, Sapporo, 060-8648 Japan; c First Department of Internal Medicine, School of Medicine, University of Occupational and Environmental Health, 1-1, Iseigaoka, Yahata-nishi-ku, Kitakyushu, Fukuoka, 807-8555, Japan; d Division of Rheumatology, Department of Internal Medicine, Keio University School of Medicine, 35 Shinanomachi, Shinjuku-ku, Tokyo 160-8582 Japan; e Department of Rheumatology and Clinical Immunology, Graduate School of Medicine, Kyoto University, 54 Shogoin-Kawahara-cho, Sakyo-ku, Kyoto, 606-8507, Japan; f Department of Rheumatology, Tokyo Medical and Dental University, 1-5-45, Yushima, Bunkyo-ku, Tokyo, 113-8519, Japan; g NTT Sapporo Medical Center and Department of Medicine II, Hokkaido University Graduate School of Medicine, Minami 1, Nishi 15, Chuo-ku, Sapporo, 060-0061, Japan

**Keywords:** Rheumatoid arthritis, Infliximab, TNF-α, Randomized controlled trials, Sustained remission, Protocol paper

## Abstract

Infliximab, an inhibitor of TNF-α, is one of the most widely used biological disease-modifying antirheumatic drugs. Recent studies indicated that baseline serum TNF-α could be considered as a key indicator for optimal dosing of infliximab for RA treatment to achieve the clinical response and its sustained remission. The Remission induction by Raising the dose of Remicade in RA (RRRR) study is an open-label, parallel group, multicenter randomized controlled trial to compare the proportions of clinical remission based on the simplified disease activity index (SDAI) after 1 year of treatment and its sustained remission rate after another 1 year between the investigational treatment strategy (for which the dose of infliximab was chosen based on the baseline serum TNF) and the standard strategy of 3 mg/kg per 8 weeks of infliximab administration in infliximab-naïve patients with RA showing an inadequate response to MTX. The primary endpoint is the proportion of patients who kept discontinuation of infliximab 1 year after discontinued infliximab at the time of 54 weeks after the first administration of infliximab. The secondary endpoints are the proportion of clinical remission based on SDAI and changes in SDAI from baseline at each time point, other clinical parameters, quality of life measures and adverse events. Target sample size of randomized patients is 400 patients in total. The main results of the RRRR study are expected to be published at the end of 2017.

## 1 Introduction

Rheumatoid arthritis (RA) is a progressive systemic inflammatory disease characterized by joint destruction and functional disability [1]. RA occurs globally in about 1.0% of the general population, with 2–4-times higher prevalence in women than in men [2]. Although the etiology of RA is not quite clear, some inflammatory cytokines such as tumor necrosis factor α (TNF-α) have been shown to play a central role in the occurrence and progression of RA [3].

Infliximab, an inhibitor of TNF-α, is one of the most widely used biological disease-modifying antirheumatic drugs (DMARDs); combined use of infliximab and methotrexate (MTX) shows clinical and radiographic benefits compared with placebo in patients inadequately controlled with therapeutic doses of MTX [4]. Because the therapeutic effects of infliximab (plus MTX) have been demonstrated in several clinical studies [5], [6], [7], [8], [9], [10], [11], the primary goal of RA treatment has shifted from the achievement of clinical remission to sustained remission without biologic DMARDs particularly in patients with RA in sustained remission [12], [13].

The first study reporting the possibility of biologic-free treatment in patients with RA was the TNF20 study [10]. This trial indicated that early treatment of RA with infliximab induces a permanent response that persists, even after discontinuation of the drug. After publication of the TNF20 study, the Behandelstrategieёn (BeSt) study evaluated biologic-free treatment in much larger cohort [8], [14]. Sixty-four percent of patients with early RA were able to discontinue infliximab and in 56% patients treated with MTX monotherapy for 2 years, low disease activity was maintained and progression of joint damage was inhibited. In established RA patients exhibiting an inadequate response to MTX, the Remission induction by Remicade in RA patients (RRR) study also examined the possibility of biologic-free remission or low disease activity [15]. The patients enrolled in the study were those who had reached and maintained a disease activity score 28 (DAS28) of less than 3.2 for more than 24 weeks with infliximab treatment and who then agreed to discontinue the treatment. Among the 102 evaluable patients who completed the study, 56 (55%) maintained low disease activity after 1 year and showed no progression in radiological damage and functional disturbance; 44 (43%) remained in clinical remission (DAS28 < 2.6).

In this context, subanalysis of the dose-escalation study of infliximab with MTX (RISING study) showed a significant interaction between baseline TNF-α and the dose of infliximab in the clinical response. Additionally, the clinical response and disease activity were significantly better when the treatment was applied at 10 mg/kg than at 3 and 6 mg/kg, with a high baseline TNF-α (baseline TNF-α values: 1.65 pg/mL or greater) [16]. To achieve the clinical response and its sustained remission, serum TNF-α could be considered as a key indicator for optimal dosing of infliximab for RA treatment.

The Remission induction by Raising the dose of Remicade in RA (RRRR) study was planned to compare the proportions of clinical remission based on the simplified disease activity index (SDAI) after 1 year of treatment and its sustained remission rate after another 1 year between the investigational treatment strategy (for which the dose of infliximab was chosen based on the baseline serum TNF) and the standard strategy of 3 mg/kg per 8 weeks of infliximab administration in infliximab-naïve patients with RA showing an inadequate response to MTX. In this study, we describe the study design and baseline characteristics of the enrolled patients.

## 2 Methods

### 2.1 Eligible patients

Patients with RA were eligible for enrollment if they had active disease equal to or greater than 6 mg MTX weekly, were 18 years of age or older at the time of enrollment, and experienced no prior infliximab use. Patients were excluded if they were taking corticosteroids at doses higher than 10 mg prednisolone equivalents/day, had an SDAI ≤11.0, had severe infections, had active tuberculosis or evidence of latent tuberculosis, were given a diagnosis of systemic lupus erythematosus or any other form of concomitant arthritis, had congestive heart failure, or were pregnant or lactating women during or 6 months after treatment. All the patients gave written informed consent in accordance with the Declaration of Helsinki, and the trial was approved by the institutional review board at each participating institution. This trial was registered with University Hospital Medical Information Network (UMIN; number UMIN000005113).

### 2.2 Study design

**Fig. 1:**
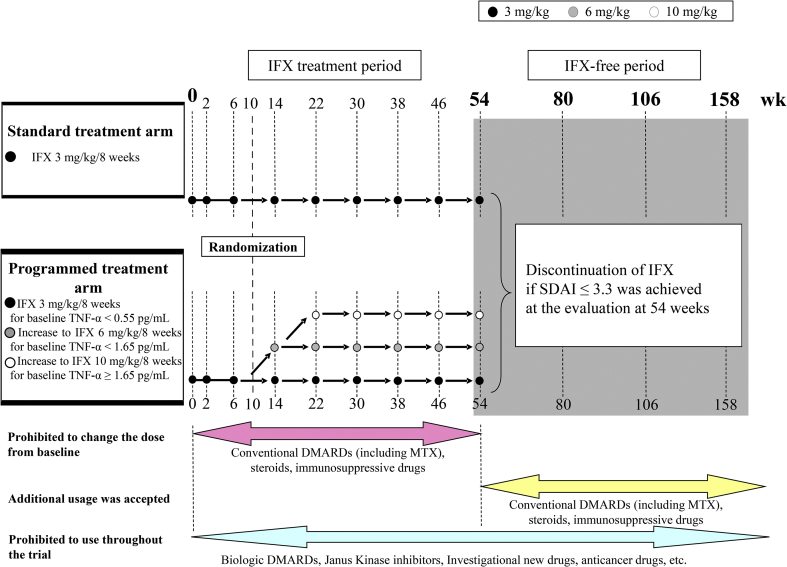
Study Design of the RRRR study.

The RRRR study was conducted as an open-label, parallel group, multicenter randomized controlled trial. Eligible patients with RA who had active disease despite taking equal to or greater than 6 mg of MTX weekly were able to participate. They were randomly assigned in a 1:1 ratio to receive either a standard treatment (standard dose of 3 mg/kg infliximab every 8 weeks) or a programmed treatment with the starting dose of infliximab based on the three categories of baseline TNF-α (low, less than 0.55 pg/mL; intermediate, 0.55 pg/mL or greater to less than 1.65 pg/mL; and high, 1.65 pg/mL or greater) in addition to baseline MTX after 10 weeks of enrollment (Fig. 1).

To ensure a balanced group design, the Clinical Research and Medical Innovation Center at Hokkaido University Hospital centrally performed the randomization using a computer-generated random number-producing algorithm. Patients were randomly assigned one to one ratio to the standard treatment arm or the programmed treatment arm with the use of permuted block (blocks size was determined randomly as four or six) within each stratum. Sixteen strata for randomization consisted of disease duration (less than 3 years or not), baseline SDAI (less than 26 or not), and baseline TNF-α (less than 0.55 pg/mL, 0.55 pg/mL or greater to less than 1.65 pg/mL, or 1.65 pg/mL or greater). Treatment allocation was blinded for the reviewer of the patients' disease, but was open for both the patients and the physicians.

Clinical response was measured using SDAI, which is a well-validated measure of composite clinical disease activity [17]. If patients had achieved an SDAI of less than or equal to 3.3 at the end of 54 weeks, they discontinued infliximab. Discontinuation of infliximab was maintained throughout follow-up until 158 weeks after enrollment unless patients showed clinical or radiologic progression.

### 2.3 Treatment plan

The treatment plans for the standard treatment arm and the programmed treatment arm are described in detail below.

#### 2.3.1 Standard treatment arm

After enrollment, patients received 3 mg/kg infliximab at 0, 2, and 6 weeks. The same dose was taken every 8 weeks after 14 weeks. If the patients showed an SDAI of less than or equal to 3.3 at 54 weeks, they discontinued infliximab.

#### 2.3.2 Programmed treatment arm

After the enrollment, the patients received 3 mg/kg infliximab at 0, 2, and 6 weeks. The dose of infliximab was selected based on baseline serum TNF-α.

⁃ If serum TNF-α was less than 0.55 pg/mL, infliximab was kept at 3 mg/kg every 8 weeks after 14 weeks.
⁃ If serum TNF-α was greater than 0.55 pg/mL to less than 1.65 pg/mL, infliximab was increased to 6 mg/kg at 14 weeks and maintained at 6 mg/kg every 8 weeks after 22 weeks.
⁃ If serum TNF-α was 1.65 pg/mL or greater, infliximab was increased to 6 mg/kg at 14 weeks and to 10 mg/kg at 22 weeks; the dose of 10 mg/kg was then administered every 8 weeks after 30 weeks. If the patients showed an SDAI ≤3.3 at 54 weeks, they discontinued infliximab.

The allocated dose could not be changed. Patients were dropped from the trial if they used biological DMARDs except for infliximab, increased the dose in the standard treatment arm, did not increase the dose in the programmed treatment arm, could not continue the treatment due to adverse events, were re-introduced infliximab after the discontinuation of infliximab, or had other reasons (e.g., withdrawal consent). During the infliximab treatment period (Fig. 1), the same dose of concomitant treatment at baseline was accepted, and dose reduction or halting of concomitant treatments was also possible if necessary.

### 2.4 Outcome measures

The primary endpoint was the proportion of patients who sustained discontinuation of infliximab 1 year after discontinuation of infliximab at the time of 54 weeks after the first administration of infliximab. The secondary endpoints were the proportion of clinical remission at the time of 54 weeks after the first administration of infliximab; the portion of patients who sustained discontinuation of infliximab at 2 years after discontinuation of infliximab; the proportion of clinical remission based on SDAI and changes in SDAI from baseline at each time point; the proportion of clinical remission based on DAS28-ESR, DAS28-CRP, and Boolean-based definitions and change in each value at each time point; radiographs of the hands, wrists, and feet, which were centrally assessed and assigned a score according to the van der Heijde modifications of the total Sharp score (modified total Sharp score); rheumatoid factor and matrix metalloproteinase-3 (MMP-3); health assessment questionnaire (HAQ) and EQ-5D; serum infliximab concentration at the time of 54 weeks after the first administration of infliximab; and adverse events. Table 1 shows the details of data collection during the trial.

**Table 1:** Data collection schedule.

Visit	Baseline	IFX trt period				IFX free period			Drop-out
		First IFX administration	Second and third IFX administration	IFX administration every 8 weeks	Last IFX administration	6 months after discontinuation	1 year after discontinuation	2 years after discontinuation	
		0 weeks	2, 6 weeks	14–46 weeks	54 weeks	80 weeks	106 weeks	158 weeks	
Informed consent	X								
Patient background	X								
TNF-α concentration	X								
IFX administration		X	X	X	X				
SDAI, DAS	X		X	X	X	X	X	X	X
RF, MMP-3	X				X		X	X	X
Radiographic assessment	X				X		X	X	X
Serum IFX concentration, ATI					X				
Commitment medications		X	X	X	X	X	X	X	X
HAQ, EuroQOL		X	X	X	X	X	X	X	X
Adverse events		X	X	X	X	X	X	X	X

### 2.5 Sample size and statistical considerations

Based on the RISING study, the proportions of clinical remission (SDAI ≤ 3.3) were assumed to be 21% and 34% for the standard treatment arm and programmed treatment arm, respectively [16]. After the discontinuation of infliximab, if we assumed that the proportion of patients who sustained discontinuation was set as 55% in the standard treatment arm and 65% in the programmed treatment arm [15], the proportions of patients who sustained discontinuation of infliximab at 1 year after a discontinuation of infliximab at the time of 54 weeks after the first administration of infliximab in the standard treatment arm and programmed treatment arm were calculated as 11.6% (= 21%*55%) and 22.1% (= 34%*65%), respectively. Based on 11.6% in the standard treatment and 22.1% in the programmed treatment arm, 199 randomized patients were needed for each treatment arm to have 80% of power at a two-sided 5% level of significance (400 patients in total). Considering the dropout rate of approximately 10% between the enrollment and the randomization, we sought to enroll 450 patients at most in the trial until the end of September 2013.

Primary analysis will be conducted based on the intention-to treat population, which included all the patients enrolled and randomized in the trial. The proportion of sustained discontinuation at 1 year after a discontinuation of infliximab at the time of 54 weeks will be compared using the Cochrane-Mantel-Haenszel test and stratification factors with disease duration (less than 3 years or not) and baseline SDAI (less than 26 or not). Risk difference of the proportion of sustained discontinuation at 1 year after a discontinuation of infliximab and its 95% confidence intervals (95% CI) will be calculated. To confirm the robustness of the primary results, the same analyses will be conducted in the population restricted to the patients who completed the planned infliximab and entered the infliximab-free period (secondary analysis population). Subgroup analysis based on the disease duration, baseline SDAI, and baseline TNF-α concentration will be planned.

For the secondary endpoints, we will conduct the same analysis for a proportion of patients with clinical remission at the time of 54 weeks and a portion of patients who sustained discontinuation of infliximab at 2 years after a discontinuation of infliximab. The proportions of clinical remission according to DAS28-ESR-, DAS28-CRP-, and Boolean-based definitions will be calculated. Changes from baseline in SDAI, DAS28, rheumatoid factor, MMP-3, HAQ, EQ-5D, and the total Sharp score will be analyzed using a mixed model for repeated measures (MMRM) [18]. Means and standard deviations will be calculated for all time points and displayed as a transition diagram. Time to discontinuation of infliximab and time until the loss of efficacy will be plotted using the Kaplan-Meier method with key survival statistics (n, n censored, 25th percentile, median, and 75th percentile). Treatment arms will be compared using log-rank tests, and hazard ratios and 95% CIs will be estimated using a Cox proportional hazards model.

Safety analysis will be conducted based on the safety population, which included all patients who enrolled in the study and received infliximab at least once. The combined results with the treatment arms will be shown before randomization and shown separately for each treatment arm after the randomization. The numbers and proportions of adverse events will be calculated. As an exploratory analysis, logistic regression analyses will be performed in order to identify the predictors of clinical remission at 52 weeks and sustained remission after 1 year.

Baseline characteristics and compliance of the treatment will be summarized using descriptive statistics (N, mean, standard deviation, median, 25th and 75th percentiles, minimum and maximum for continuous data, and N [%] for categorical data) for each treatment arm and overall for each safety population and the intention-to treat population. All *P* values calculated in the analysis will be one-sided and will not be adjusted for multiple testing since no interim analysis is planned. *P* values of less than 0.025 will be considered to indicate statistical significance. We will use SAS version 7.4 (SAS Institute, Cary, NC, USA) for all the analyses.

## 3 Results

From April 2011 to September 2013, 413 patients with RA agreed to participate, and 405 patients were enrolled in the RRRR study. Eight patients were not enrolled because they did not meet the eligibility criteria. Patient characteristics of the enrolled patients are presented in Table 2. Mean age was 57.7 years, and 320 (79%) patients were female. Mean disease duration was 56.4 months, and the number of patients who had high disease activity based on SDAI (SDAI > 26) was 180 (44.4%). The mean DAS28 (CRP) and DAS28 (ESR) scores were 4.7 (standard deviation = 1.2) and 5.4 (standard deviation = 1.2), respectively, and these are comparable to the values obtained in our previous observational study [15]. The distribution of serum TNF-α was also shown. The numbers of patients for each baseline serum TNF-α category (less than 0.55 pg/mL, 0.55 pg/mL or greater to less than 1.65 pg/mL, or 1.65 pg/mL or greater) were 123 (30.4%), 152 (37.6%), and 129 (31.9%), respectively. The percentage of baseline serum TNF-α 1.65 pg/mL or greater in the RRRR Study was higher than that in the RISING Study [16].

**Table 2:** Baseline characteristics of patients enrolled in RRRR study.

	N	%
**Total**	405	100.0
Sex		
Female	320	79.0
History of surgery		
Yes	26	6.4
Complication		
Yes	219	54.1
DMARDs use		
Yes	163	40.2
NSAIDs use		
Yes	283	69.9
SDAI >26		
Yes	180	44.4
DAS28 (CRP) ≥ 5.1		
Yes	132	32.6
DAS28 (ESR) ≥ 5.1		
Yes	237	58.5
TNF-α concentration		
<0.55 pg/mL	123	30.4
0.55 to < 1.65 pg/mL	152	37.6
>1.65 pg/mL	129	31.9

	Mean	SD	Min	Median	Max
Age (years)	57.7	13.1	20	59	84
BMI (kg/m^2^)	22.3	3.4	14.0	21.8	39.0
Duration (month)	56.4	86.0	1	19	696
Dose of MTX (mg)	10.9	3.2	6.0	10.0	17.5
Tender joint count	8.3	6.4	0	6	28
Swollen joint count	7.6	5.3	0	6	28
Rheumatoid factor	121.7	232.3	0.0	49.9	2301.0
CRP (mg/dL)	2.2	4.0	0.0	1.0	58.0
Erythrocyte sedimentation rate	47.0	31.3	0.1	40.0	160.0
Patient's global assessment of disease	51.7	24.5	2	52.0	100
Physician's global assessment of disease	49.7	20.8	5	50.0	100
MMP-3	210.7	227.5	10.0	131.0	1620.0
TNF-α	5.0	35.6	0.55	1.1	497.0
SDAI	28.2	14.2	11.1	24.4	108.1
DAS28 (CRP)	4.7	1.2	1.7	4.7	8.1
DAS28 (ESR)	5.4	1.2	0.1	5.4	8.6
HAQ	1.1	0.8	0.0	1.0	3.0

## 4 Discussion

In this protocol paper, we have described the details of the study design, analysis plan, and baseline characteristics of patients enrolled in the RRRR Study. Although several previous studies have provided the evidence of the possibility of sustained “biologic-free remission” with no functional or radiographic progression in patients with early RA after treatment with combination of TNF inhibitors and MTX [8], [9], [10], [19], [20], [21], [22], [23], the efficacy results are less convincing for established patients with RA who have inadequate responses to MTX. Smolen et al. evaluated whether low disease activity would be sustained with reduced doses or withdrawal of Etanercept in patients with moderately active RA (PRESERVE Study) [24]. In their study, conventional or reduced doses of Etanercept with MTX in patients with established RA more effectively maintained low disease activity than did MTX alone after withdrawal of Etanercept. In contrast, Tanaka et al. investigated the possibility of discontinuing adalimumab for 1 year without flaring (DAS28-ESR ≥ 3.2), and they found that the proportions of patients who sustained a DAS28-ESR score of less than 2.6 or less than 3.2 for 1 year were not significantly different between the ADA discontinuation group and the ADA continuation group if patients acquired deep remission (DAS28-ESR ≤ 1.98) [25]. The latest American College of Rheumatology Guideline does not recommend to discontinue all RA therapies for patients with established RA [12] even if the patient's disease is in remission.

The RRRR study is the first randomized trial to compare the efficacy of the treatment strategy between the investigational treatment in which the dose of infliximab was decided based on the baseline serum TNF, and the standard dose (3 mg/kg) of infliximab in established patients with RA exhibiting an inadequate response to MTX. After the results of the trial are published, we may be able to answer several clinical questions as follows. First question was if it is really possible for established patients with RA to discontinue infliximab with sustained clinical remission after the achievement of a SDAI of less than or equal to 3.3 at 54 weeks. Second question was whether the baseline serum TNF-α is a biomarker of therapeutic response to optimize choice of treatment strategy. The final question was, what the baseline predictive factor was for clinical remission and whether we could identify baseline and other biomarkers (including serum infliximab concentration at the time of 54 weeks) for sustained remission. Considering the potential safety issues and the economic burden caused by the use of biologic DMARDs, all of the above clinical questions are important when we consider the possibility of discontinuation of biologic DMARDs after achieving remission. The main results of the RRRR study are expected to be published at the end of 2017.

## Conflicts of interest

Y. Tanaka has received consulting fees, speaking fees, and/or honoraria from Abbvie, Chugai, Daiichi-Sankyo, Bristol-Myers, Mitsubishi-Tanabe, Astellas, Takeda, Pfizer, Teijin, Asahi-kasei, YL Biologics, Sanofi, Janssen, Eli Lilly, and GlaxoSmithKline and has received research grants from Mitsubishi-Tanabe, Takeda, Daiichi-Sankyo, Chugai, Bristol-Myers, MSD, Astellas, Abbvie, and Eisai.

T. Takeuchi has received grants from Astellas Pharma Inc, Bristol–Myers K.K., Chugai Pharmaceutical Co, Ltd., Daiichi Sankyo Co., Ltd., Eisai Co., Ltd., AYUMI Pharmaceutical Corporation, Takeda Pharmaceutical Co., Ltd., Teijin Pharma Ltd., AbbVie GK, Asahikasei Pharma Corp., and Taisho Toyama Pharmaceutical Co., Ltd. Speaking fees from AbbVie GK., Bristol–Myers K.K., Chugai Pharmaceutical Co,. Ltd., Eisai Co., Ltd., Janssen Pharmaceutical K.K., Mitsubishi Tanabe Pharma Co., Pfizer Japan Inc., and Takeda Pharmaceutical Co., Ltd., Astellas Pharma Inc, and Diaichi Sankyo Co.,Ltd., Celtrion, Nipponkayaku Co. Ltd. Consultant fees from Astra Zeneca K.K., Eli Lilly Japan K.K., Novartis Pharma K.K., Mitsubishi Tanabe Pharma Co., and Asahi Kasei Medical K.K., abbivie GK, Daiichi Sankyo Co.,Ltd., Bristol–Myers K.K., Nipponkayaku Co. Ltd, Janssen Pharmaceutical K.K., Merck Serono Co.,Ltd., Takeda Pharmaceutical Co., Ltd., Astellas Pharma Inc,. Pfizer Japan Inc..

T. Mimori received research grants and/or speaker's bureaus from Actelion, Asahi Kasei Pharma, Astellas, Ayumi, Bristol-Myers Squibb, Chugai, Daiichi Sankyo, Eisai, Mitsubishi-Tanabe, MSD, Nippon-Kayaku, and Takeda.

T Koike has received speakers bureau from AbbVie, Asuka Pharma, Astellas Pharma, Bristol-Myers, Chugai Pharmaceutical, Daiichi Sankyo, Eisai, Mitsubishi Tanabe Pharma, Pfizer Japan, Teijin Pharma, UCB Pharma. Consultant fee from Bristol-Myers, Eli Lilly Japan, Pfizer Japan, Daiichi Sankyo, Sanofi.

Other members have no conflict of interest to be declared associated with this publication.
